# The Brief Case: *Acanthamoeba* meningoencephalitis in a transplant recipient

**DOI:** 10.1128/jcm.00350-24

**Published:** 2025-02-19

**Authors:** Vanessa M. Kung, Lilian Vargas Barahona, Esther Benamu Sultan, Poornima Ramanan, B. K. Kleinschmidt-DeMasters, Bruce D. McCollister, Nancy E. Madinger

**Affiliations:** 1 Division of Infectious Diseases, University of Pittsburgh 271847 , Pittsburgh, Pennsylvania, USA; 2 Pittsburgh VA Hospital System, Pittsburgh, Pennsylvania, USA; 3 Division of Infectious Diseases, Creighton University 12282 , Omaha, Nebraska, USA; 4 Division of Infectious Diseases, North Shore University Hospital 24945 , Manhasset, New York, USA; 5 Division of Infectious Diseases, Oregon Health & Science University 6684 , Portland, Oregon, USA; 6 Department of Pathology, University of Colorado Anschutz Medical Campus 197284 , Aurora, Colorado, USA; 7 Department of Neurosurgery, University of Colorado Anschutz Medical Campus 156150 , Aurora, Colorado, USA; 8 Division of Infectious Diseases, University of Colorado 228891 , Aurora, Colorado, USA; Boston Children's Hospital, Boston, Massachusetts, USA

**Keywords:** *Acanthamoeba*, free-living amebae, meningoencephalitis, brain abscess, granulomatous amebic encephalitis (GAE), transplant infectious diseases

## CASE

A 64-year-old male, who had received a single lung transplant for interstitial lung disease 3 years prior to presentation, on stable immunosuppression with tacrolimus and prednisone, presented to a hospital with a 20-day history of headache and intermittent altered mentation. His post-transplant course had been complicated by pulmonary aspergillosis diagnosed 1 year prior, for which he was on voriconazole. The patient lived in Texas and traveled frequently to Mexico. On admission, he had a seizure.

The patient was afebrile, normotensive, and required 1 L/min of oxygen by nasal cannula. His white blood cell count was normal and without neutropenia or lymphopenia. The comprehensive metabolic panel was within normal limits. Brain magnetic resonance imaging (MRI) without contrast showed a T2 signal along the bilateral frontal lobes with mildly increased diffusion. Chest computed tomography showed ground glass opacities throughout the transplanted left lung and fibrotic native right lung and known mycetomas. Cytomegalovirus (CMV) viremia was detected. Blood aspergillus galactomannan (index 2.66) and 1,3-beta-D-glucan (230 pg/ml) were positive. The cerebrospinal fluid (CSF) analysis showed lymphocytic pleocytosis, with 36 × 10^6^ /L nucleated cells, 98% lymphocytes, elevated protein 85 mg/dL, and normal glucose 44 mg/dL (serum glucose 181 mg/dL). Opening pressure was not recorded. The patient was started on liposomal amphotericin B, intravenous ganciclovir, CMV immunoglobulin, and empiric broad-spectrum antibiotics. Extensive work-up of CSF, including viral polymerase chain reaction (PCR) for CMV, herpes simplex virus, varicella zoster virus, Epstein Barr virus, JC virus, and adenovirus, was negative. Cryptococcal antigen in serum and CSF, histoplasma urine and serum antigens, *Coccidioides* antibody panel, *Mycobacterium tuberculosis* PCR of CSF, serum *Treponema pallidum* antibodies, and *Toxoplasma gondii* PCR of serum and CSF were negative.

**Fig 1:**
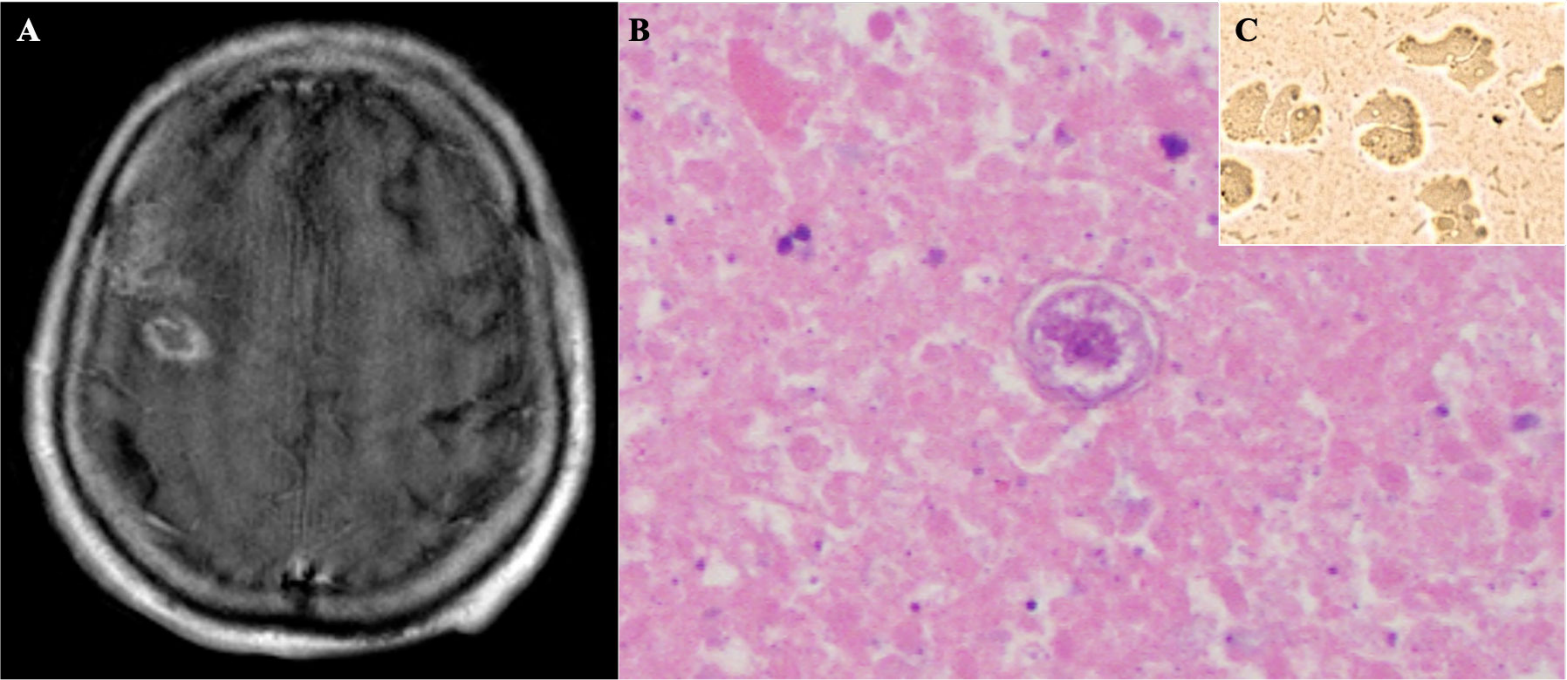
**(A)** MRI with contrast of the brain, showing a ring-enhancing lesion in the right frontal lobe. **(B)** Histopathologic exam with H&E stain of a right frontal lobe brain lesion, showing an *Acanthamoeba* cyst with a thick crenated wall, a single nucleus with prominent karyosome, and surrounding necrotic brain tissue. **(C)** *Acanthamoeba* culture of brain tissue was positive for *Acanthamoeba* trophozoites.

The patient developed a worsening headache. The repeat CSF analysis after 1 week showed persistent lymphocytic pleocytosis. CSF beta-D-glucan was negative. Free-living amebae (*Naegleria fowleri*, at least 20 validated *Acanthamoeba* strains, and *Balamuthia mandrillaris*) detection by PCR of CSF was negative (Mayo Clinic Laboratories, Rochester, MN). CSF broad-range PCR studies for bacteria and fungi were negative (University of Washington, Seattle, WA). Additionally, *Echinococcus, Taenia solium*, and *Strongyloides* antibodies in serum were negative. Bronchoalveolar lavage fluid cultures grew *Aspergillus flavus*, and there was concern that the brain lesions were aspergillomas. After several weeks of continued amphotericin, there was no change in clinical condition. An MRI brain with contrast showed ring-enhancing lesions in the bifrontal lobes (Fig. 1A), and a brain biopsy was performed on hospital day 30. Bacterial, fungal, and AFB cultures of brain tissue were negative.

Right frontal brain lesion biopsy revealed dense chronic inflammatory infiltrate, with adjacent necrosis and organisms with thick translucent refractile walls and single nucleus (Fig. 1B), consistent with amebic encephalitis. *Acanthamoeba* culture of brain tissue was positive (Fig. 1C). PCR of brain tissue was positive for *Acanthamoeba* and negative for *Balamuthia* and *Naegleria* (Mayo Clinic Laboratories). Metronidazole, azithromycin, pentamidine, miltefosine, and voriconazole were started. Additional surgical resection of abscesses was recommended; however, this did not align with the patient’s goals of care. He was discharged to home hospice and died shortly thereafter.

## DISCUSSION

Free-living amebae, including *Naegleria fowleri, Acanthamoeba* spp. (e.g., *A. castellanii, A. culbertsoni, A. divionensis*, *A. healyi*, and *A*. *polyphaga*), *Balamuthia mandrillaris,* and *Sappinia pedata*, have been recognized in recent decades as etiologies of severe brain infections. *Naegleria* is typically fulminant and mostly described in young and otherwise-healthy individuals. Though eventually equally fatal, *Acanthamoeba* and *Balamuthia* tend to have more indolent courses and are opportunistic infections in immunocompromised hosts (1). Free-living amebae are parasites acquired from the environment, which infect humans through skin or mucosal contact with soil or water. Most known for its association with keratitis in contact lens users, *Acanthamoeba* can cause localized or disseminated disease in immunocompromised patients. Both the diagnosis and treatment of *Acanthamoeba* meningoencephalitis remain challenging, and reported case fatality rates range from 70% to over 90% (1). We report a fatal case of a lung transplant recipient with *Acanthamoeba* meningoencephalitis, illustrating how the timely diagnosis and effective treatment of *Acanthamoeba* meningoencephalitis remain challenging in clinical medicine.

Diagnosis of free-living amebae is frequently delayed or even identified post-mortem. Clinically, *Acanthamoeba* and *Balamuthia* should be on the differential diagnoses of subacute presentations of meningoencephalitis, particularly for immunocompromised hosts. Culturing of free-living amebae is a diagnostic tool that is relatively affordable for microbiology laboratories but may depend upon invasive tissue sampling, and laboratory ability to grow amebae in described conditions. *Acanthamoeba* can be cultured from the brain and other tissue samples using specific conditions (that do not support the growth of more fastidious *Balamuthia*); for instance, monoxenic culture on non-nutrient agar seeded with a lawn of *Escherichia coli*, and then morphologically identified by microscopic examination (1, 2). On light microscopy, *Acanthamoeba* and *Balamuthia* are easily distinguishable from *Naegleria* (Table 1). Compared to *Naegleria, Acanthamoeba* trophozoites are larger, have more numerous and spinier pseudopodia (“acanthopodia”), are less motile at room temperature, and cannot enter a flagellate stage. *Acanthamoeba* cysts can form in human tissue, unlike *Naegleria* cysts. *Acanthamoeba* and *Balamuthia* are nearly indistinguishable on light microscopy but electron microscopy can be used to identify that *Acanthamoeba* cysts have two-layered walls with pores, whereas *Balamuthia* cysts have three-layered walls without pores. Immunofluorescence, immunohistochemistry, or nucleic acid detection techniques can also be used to identify *Acanthamoeba* from tissue samples. Our patient did not have keratitis nor other mucocutaneous manifestations of the infection, which are more accessible than brain tissue for biopsy and could provide an earlier diagnosis.

**TABLE 1:** Comparison of free-living amebic CNS infections

	*Naegleria fowleri*	*Acanthamoeba* spp.	*Balamuthia mandrillaris*
Epidemiology and clinical features			
Host	Young and healthy	Immunocompromised	Immunocompromised, LatinX
Clinical presentation	Fulminant, primary amebic meningoencephalitis (PAM)	Subacute, granulomatous amebic encephalitis (GAE)	Subacute, granulomatous amebic encephalitis (GAE)
Brain imaging	Nonspecific	Space-occupying lesions	Space-occupying lesions
CSF wet mount	Amebae positive	Amebae rarely seen	Amebae rarely seen
Morphology of amebae			
Trophozoite	Smaller (10–40 µm), with few smooth pseudopodia; motile at 22–25°C and 35–37°C; flagellate stage exists; replicate by promitosis; found in human tissue and CSF	Larger (10–45 µm), with many spiny pseudopodia (“acanthopodia”); sluggish at 22–25°C and motile at 35–37°C; no flagellate stage; replicate by metamitosis; found in human tissue	Larger (10–60 µm), with slightly broader pseudopodia than those of *Acanthamoeba*; no flagellate stage; replicate by metamitosis; found in human tissue
Cyst	Smaller (7–15 µm); thin-walled (one- layered); *not* found in human tissue	Larger (10–25 µm), thick-walled (two- layered wall with pores); found in human tissue	Larger (10–25 µm), thick-walled (three-layered wall without pores); found in human tissue

Prior to brain tissue biopsy, CSF studies can narrow the differential diagnosis. In contrast to its utility in the identification of *Naegleria*, wet mount examination of CSF for *Acanthamoeba* and *Balamuthia* is generally negative, underscoring the need for other diagnostic methods for these infections (1). Real-time polymerase change reaction (RT-PCR) targeting 18S rRNA gene sequences, for the detection of *Naegleria, Acanthamoeba*, and *Balamuthia* infections, is available in a limited number of reference laboratories in the United States. In this report, we emphasize that even state-of-the-art testing of CSF with RT-PCR can be negative for *Acanthamoeba* and, thus, cannot rule out *Acanthamoeba* meningoencephalitis. This is consistent with other literature reports that *Acanthamoeba* is rarely detected by PCR of CSF. Perhaps *Acanthamoeba* is transiently present in the CSF, or there is a low burden of organisms in the CSF by the time patients present clinically with brain abscesses. There are case reports demonstrating that *Naegleria* and interestingly *Balamuthia* can be detected in CSF using metagenomic next-generation sequencing of cell-free DNA (cfDNA), and more data are needed to understand the performance of cfDNA testing of CSF or plasma for the detection of free-living amebae (3). A brain tissue sample was required in our case to detect *Acanthamoeba* by RT-PCR, highlighting the importance of tissue biopsy for patients with immunocompromising conditions.

There is currently significant variability in the recommendations for the management of *Acanthamoeba* meningoencephalitis, and the Infectious Diseases Society of America (IDSA) guidelines were last updated in 2008. Curative treatment may require a combination of surgical excision and antimicrobial therapy. Few case reports have been published on transplant recipients who survived *Acanthamoeba* meningoencephalitis or dissemination (4–6). The optimal antimicrobial regimen is currently unknown, and recommendations have generally been based on *in vitro* data and case reports on patients who survived. The efficacy and adverse effects of regimens can be confounded by agents that were intended for empiric broad coverage of other, more common etiologies of ring-enhancing brain lesions. Choosing an antimicrobial regimen can additionally be complicated by the presence of co-infections, particularly as *Acanthamoeba* can act as a “Trojan horse” and harbor intracellular bacteria and other organisms (1). For our patient, with guidance from experts at the Centers for Disease Control and Prevention (CDC), we recommended a combination of miltefosine, azole, pentamidine, flucytosine, and sulfadiazine. Among these medications, miltefosine is increasingly regarded as the vital agent, and case reports have been published on cures achieved with miltefosine-based regimens. Miltefosine is an alkylphosphocholine, with potential mechanisms of action including interactions with parasite cell membranes and the inhibition of cytochrome c oxidase.

The few published reports of transplant recipients who survived *Acanthamoeba* meningoencephalitis or dissemination have commonalities in management with surgical debridement (5, 6) and/or miltefosine-containing regimens (4, 5). The first case, which occurred before miltefosine was available for patients with free-living amebic infections, was a 41-year-old male, who had received a liver transplant 14 months prior to presentation, diagnosed with *Acanthamoeba* meningoencephalitis using brain tissue biopsy and immunohistochemistry 79 days after presentation and was cured with a left frontal lobectomy (6). Next, a 60-year-old female, who presented 5 months after a heart transplant with paranasal sinus symptoms, was diagnosed 2 months after symptom onset with disseminated *Acanthamoeba* infection (*Acanthamoeba* was identified on sinus tissue, skin nodule, and bone biopsies, using histopathologic exam, immunofluorescence, and PCR) and was cured with repeated surgical debridement and a 6-month course of miltefosine, with fluconazole and flucytosine (5). Most recently, a 32-year-old male presented 5 months after an allogeneic hematopoietic stem cell transplant (for chemotherapy-refractory Hodgkin lymphoma) complicated by skin graft-versus-host-disease, was diagnosed with *Acanthamoeba* meningoencephalitis within days of presentation by brain tissue histopathologic exam and PCR, and was cured without surgical debridement, with a 5-month course of miltefosine, at times with other antimicrobials like fluconazole (4 months), pentamidine (21 days), and trimethoprim-sulfamethoxazole (28 days) (4). Further studies on host factors, the virulence of different *Acanthamoeba* strains, and management options, in combination with earlier diagnoses of this infection, may lead to the optimization of treatment strategies.

Infections with free-living amebae should remain in the differential diagnosis of subacute meningoencephalitis in immunocompromised patients. While CSF PCR for *Acanthamoeba*, *Balamuthia* and *Naegleria* may be helpful, a negative test does not rule out the infection. Biopsy of brain lesions must be considered for the timely diagnosis of free-living amebic infections.

## SELF-ASSESSMENT QUESTIONS

1. Meningoencephalitis with the following free-living amebae is more likely to result in death within days.
  - *Naegleria fowleri*
  - *Acanthamoeba* species
  - *Balamuthia mandrillaris*
  - *Sappinia pedata*
2. Which of the following studies is often needed to make a definitive diagnosis of *Acanthamoeba* meningoencephalitis?
  - Serology
  - Lumbar puncture
  - Brain tissue biopsy
  - Eye cornea scrapings
3. Treatment of *Acanthamoeba* meningoencephalitis likely requires which of the following management components?
  - Antimicrobial regimen including liposomal amphotericin B
  - Antimicrobial regimen including miltefosine
  - Serial lumbar punctures for intracranial pressure management
  - Surgical resection only

## ANSWERS TO SELF-ASSESSMENT QUESTIONS

1. Meningoencephalitis with the following free-living amebae is more likely to result in death within days.
  - *Naegleria fowleri*
  - *Acanthamoeba* species
  - *Balamuthia mandrillaris*
  - *Sappinia pedata*

a. *Naegleria* infections are fulminant, whereas *Acanthamoeba*, *Balamuthia*, and *Sappinia* infections are typically subacute.

- Which of the following studies is often needed to make a definitive diagnosis of *Acanthamoeba* meningoencephalitis?
  - Serology
  - Lumbar puncture
  - Brain tissue biopsy
  - Eye cornea scrapings

c. This case demonstrates that brain tissue biopsy is necessary for diagnosis, and even PCR of CSF can be negative for *Acanthamoeba* meningoencephalitis. There are no serologic tests for *Acanthamoeba*. Cornea scrapings can diagnose *Acanthamoeba* keratitis but not meningoencephalitis.

- Treatment of *Acanthamoeba* meningoencephalitis likely requires which of the following management components?
  - Antimicrobial regimen including liposomal amphotericin B
  - Antimicrobial regimen including miltefosine
  - Serial lumbar punctures for intracranial pressure management
  - Surgical resection only

b. Miltefosine-based antimicrobial regimens in combination with surgical resection are the current foundations of treatment. Amphotericin B has poor *in vitro* activity against *Acanthamoeba*.

**TAKE-HOME POINTS**

- Free-living amebae can be etiologies of subacute meningoencephalitis in immunocompromised patients.
- Brain tissue studies (PCR, culture, and histopathologic exam) are usually needed to diagnose *Acanthamoeba* meningoencephalitis, as CSF studies (including PCR) are less sensitive.
- Curative treatment of *Acanthamoeba* meningoencephalitis likely requires an antimicrobial regimen containing miltefosine and surgical debridement.
